# Growth Condition-Oriented Defect Engineering for Changes in Au–ZnO Contact Behavior from Schottky to Ohmic and Vice Versa

**DOI:** 10.3390/nano8120980

**Published:** 2018-11-27

**Authors:** Abu ul Hassan Sarwar Rana, Hyun-Seok Kim

**Affiliations:** Division of Electronics and Electrical Engineering, Dongguk University-Seoul, Seoul 04620, Korea; abulhassan.rana@dongguk.edu

**Keywords:** ZnO, metal-semiconductor contact, crystal defects, nanorod, microwave, oxygen plasma treatment

## Abstract

ZnO has the built-in characteristics of both ionic and covalent compound semiconductors, which makes the metal–ZnO carrier transport mechanism quite intricate. The growth mechanism-centric change in ZnO defect density and carrier concentration also makes the contact formation and behavior unpredictable. This study investigates the uncertainty in Au–ZnO contact behavior for application-oriented research and the development on ZnO nanostructures. Herein, we explain the phenomenon for how Au–ZnO contact could be rectifying or non-rectifying. Growth method-dependent defect engineering was exploited to explain the change in Schottky barrier heights at the Au–ZnO interface, and the change in device characteristics from Schottky to Ohmic and vice versa. The ZnO nanorods were fabricated via aqueous chemical growth (ACG) and microwave-assisted growth (MAG) methods. For further investigations, one ACG sample was doped with Ga, and another was subjected to oxygen plasma treatment (OPT). The ACG and Ga-doped ACG samples showed a quasi-Ohmic and Ohmic behavior, respectively, because of a high surface and subsurface level donor defect-centric Schottky barrier pinning at the Au–ZnO interface. However, the ACG-OPT and MAG samples showed a more pronounced Schottky contact because of the presence of low defect-centric carrier concentration via MAG, and the removal of the surface accumulation layer via the OPT process.

## 1. Introduction

ZnO has been emerged as an enticing semiconductor material in the research and development on wide bandgap optoelectronic and nanoelectronic applications [1,2,3,4]. At ambient conditions, it has a high exciton binding energy of 60 meV and a direct bandgap of 3.37 eV, which make it an alluring candidate for the semiconductor industry. Its worth is further highlighted because of the formation of different polymorphic shapes, including ZnO nanorods (ZNRs), nanoflowers, nanotubes, nanoparticles, nanotetrapods, nanostars, and many more, by judiciously controlling the growth conditions [5,6,7,8]. Depending upon the formation of multiple nanostructured shapes, it has potential applications in the realm of nanoelectronics, optoelectronics, nanoscale lasers, toxic gas sensors, and ultraviolet photodetectors [9,10,11,12,13,14,15].

The metal contact formation of any semiconductor is one of the most important and inevitable processes for device fabrication. For instance, Ohmic contacts to ZnO are important for various electronic and optoelectronic applications, such as solar cells, field effect transistors, and light-emitting diodes. Similarly, Schottky contacts to ZnO are important for applications in the realm of gas and chemical sensors and photodetectors, where the Schottky barrier at the interface plays a significant role in determining the device efficiency [16,17]. As per the Schottky–Mott model, metals with high work functions are prime candidates for making Schottky contacts to semiconductors [18]. However, for ZnO, it has already been argued that the metal should have low oxygen affinity, to prevent oxide layer formation at the metal–ZnO interface, which leaves us with the choice of Pt, Pd, and Au being inert to oxidation [19]. The theoretical Au–ZnO contact should be a Schottky contact according to the classical Schottky model:

(1) $$\varphi_{SB}=\varphi_{Au}-\chi_{ZnO}$$

where $\varphi_{SB}$ is the Schottky barrier height, $\varphi_{Au}$ (5.1 eV) is the Au work function, and $\chi_{ZnO}$ (4.2 eV) is the ZnO electron affinity. The theoretical $\varphi_{SB}$ calculated via Equation (1) is 0.9 eV. However, Au–ZnO contacts show disparities to the results calculated via the classical Schottky model, because the experimental calculations not only show different Schottky barrier heights, but also an Ohmic contact to ZnO because of its intricate growth chemistry and defect formation.

Herein, the practical reasons to the above mentioned Au–ZnO contact unpredictability are addressed via experimentation and reasoning. Au is a mandatory contact to ZnO, because of its non-oxidizing nature. Au is an inert metal, where some of the Ohmic metals, such as Ag, form an oxide layer on the metal–semiconductor surface. Hence, Au is preferred upon Ag for contact formation. However, Au makes a Schottky contact, and some devices use Ohmic contacts for their operation. This study propounds how Au could be used as both Schottky and Ohmic contacts for ZnO device characteristics, and why the Au–ZnO contact behavior is unpredictable at times.

The prevailing Au–ZnO contact disparities were explained under the ambit of complex ZnO growth chemistry, crystal quality, and defect engineering. We found that the unpredictability in Au–ZnO contact formation lies in the growth method-dependent defect engineering, which is still a matter of debate in research and development on ZnO. Four different ZNR samples, grown with aqueous chemical growth (ACG), Ga-doped ACG, oxygen plasma-treated (OPT) ACG, and microwave-assisted growth (MAG), were considered. The defect engineering was monitored by studying the ZNR crystalline, structural, and optical characteristics. Also, the defect engineering-centric Schottky barrier heights and Au–ZnO contact behaviors were predicted by studying the current–voltage (*I*-*V*) and Hall-effect measurement results. It was found that, depending upon the defect engineering-centric change in majority carrier concentration, the ACG and Ga-doped ACG samples showed a quasi-Ohmic and complete Ohmic contact to ZnO, respectively, while the OPT-ACG and MAG samples showed Schottky contacts to ZnO. We believe that the propounded results would be very beneficial for the application-oriented research and development on ZnO.

## 2. Materials and Methods

**Figure 1:**
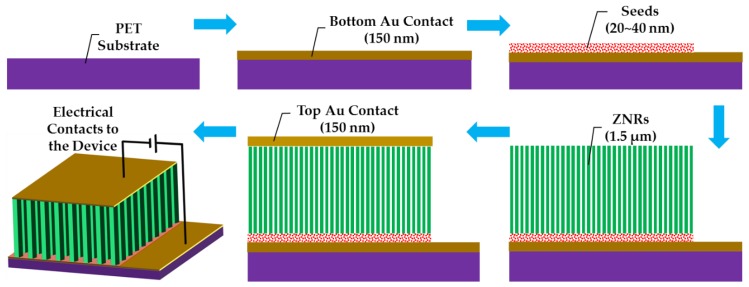
Step-by-step schematic representation of the device fabrication.

Flexible polyethylene terephthalate (PET) was used as a substrate for ZNR growth and device testing. The substrates were cleaned with acetone, isopropyl alcohol, and deionized (DI) water, and then dried with nitrogen gas. The device patterns were defined by spin coating positive photoresist (AZ 5214 E) and photolithography. The bottom electrode was deposited by evaporating 150 nm Au thin film via e-beam metal evaporation and lift-off processes. The seed layer-assisted ZNRs were directly grown onto the bottom electrode. ZNRs were fabricated via the most facile and widely accepted ACG and MAG methods, where the details of the methods are already explained in our previous reports [20,21]. A thin film seed layer deposition is necessary for vertical ZNR growth [22]. The homogeneous seed solution was made by mixing 22 mM zinc acetate dihydrate (Zn(CH_3_COO)_2_·2H_2_O) (MW 219.51 g/mol) in n-propanol (C_3_H_8_O) (M_W_ 60.10 g/mol). The seed solution was spin coated twice on the PET substrate surface, which were then annealed at 70 °C for 2 min for the first coating and at 100 °C for 2 hr for the second coating. The growth solution was made by stirring 50 mM zinc nitrate hexahydrate (Zn(NO_3_)_2_·6H_2_O) (M_W_ 297.48 g/mol) and 50 mM methenamine (C_6_H_12_N_4_) (M_W_ 140.186 g/mol) in DI water. After one hour of continuous stirring, the Au-coated seeded PET substrates were immersed in the solution autoclaves, which were then placed on a hotplate at 75 °C for 12 hr for ACG growth, and in a domestic 850 watt (2.7 GHz) microwave oven for the MAG process. One of the ACG samples was doped with 5% Ga content. The details of the doping process are explained in our previous reports [23,24]. In short, gallium nitrate hydrate (Ga(NO_3_)_3_·xH_2_O) (M_W_ 255.74 anhydrous basis) was mixed with the 50 mM zinc nitride hexahydrate and methenamine growth solution before putting the solution autoclave onto the hot plate. To probe the effects of surface and subsurface cleaning, another ACG sample was subjected to OPT with a flow rate of 100 sccm at 100 W. Afterward, the 150 nm top Au contact was directly evaporated via e-beam and lift-off processes on the ACG, Ga-doped ACG, OPT-ACG, and MAG ZNRs without any further surface processing. The final device structures for all the aforementioned devices were the same, which is presented in Schematic Figure 1.

To look at the detailed morphology, the ZNRs were characterized with scanning electron microscopy (SEM: Hitachi S-4800, Hitachi, Tokyo, Japan). The structural and crystalline properties were probed with X-ray diffraction (XRD: Rigaku Ultima IV, Rigaku, Tokyo, Japan) operating at a Cu Kα radiation of λ = 0.15418 nm. The defect centers and optical characteristics were analyzed with photoluminescence (PL: Accent RPM 2000, Accent Optical Technologies Inc., Bend, OR, USA) spectroscopy with a PL range of 300–700 nm at room temperature. The ZNR carrier concentrations were found with a Hall-effect measurement system (ECOPIA: AHT55T5, Ecopia Corporation, Anyang, Gyeonggi-do, South Korea). The *I*-*V* characteristics were measured with Keithley 2410 measurement setup (Keithley Instruments, Solon, OH, USA) at ambient conditions. The photolithographic patterns were defined with Karl Suss MA 6 mask aligner (SUSS MicroTec, Munich, Germany), and the Au contacts were deposited via e-beam metal evaporator (Korea Vacuum: KVE-T5560, Korea Vacuum Tech., Gimpo, Gyeonggi-do, South Korea) at high vacuum conditions.

## 3. Results and Discussion

### 3.1. ZnO Surface Morphology

**Figure 2:**
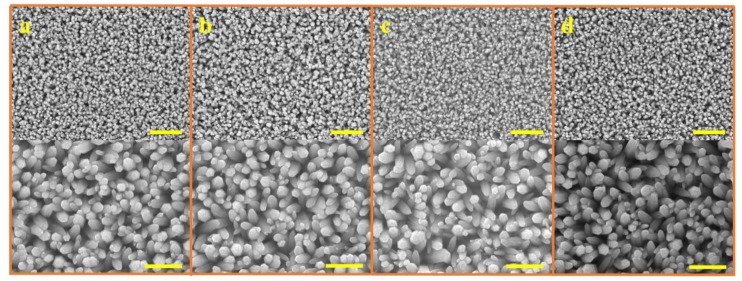
SEM images of ZNRs grown with (**a**) ACG, (**b**) Ga-doped ACG, (**c**) OPT-ACG, and (**d**) MAG methods. The scale bars in the top and bottom images correspond to 3 and 1 microns, respectively.

Figure 2 shows that the ACG, Ga-doped ACG, OPT-ACG, and MAG ZNRs have almost the same sizes and dimensions, which were deliberately kept constant to standardize the surface-to-volume ratio of the samples grown with different techniques. In this particular backdrop, the morphology-induced surface-to-volume ratio standardization is very important, so that the real essence of Au–ZnO contact behavior can be probed because of defect engineering-induced change in Schottky barrier heights, rather than the change in morphology. All of the ZNRs had the same diameter of ~200 nm, and the ZNR lengths were fixed to ~1.5 µm. The MAG solution replacement method was used to control the MAG ZNR morphology, where the 50 mM solution was replaced three times to obtain the desired results [21].

### 3.2. ZNR Structural and Crystalline Properties

**Figure 3:**
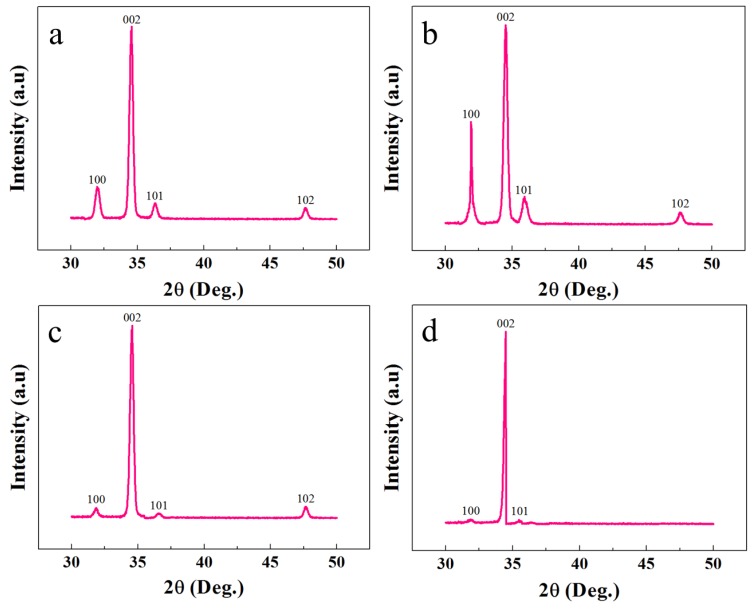
XRD patterns of ZNRs grown with (**a**) ACG, (**b**) Ga-doped ACG, (**c**) OPT-ACG, and (**d**) MAG methods.

XRD analysis was exploited to study the crystalline and structural characteristics of the ZNRs. Figure 3 shows the XRD responses of all the samples. It is confirmed that all the samples showed the typical hexagonal wurtzite ZnO character with all the peaks along the pertinent ZnO phase. In all of the samples, the tallest peak along the 002 direction confirms the ZNR vertical orientation perpendicular to the substrate surface. The XRD phase-dependent best ZNR orientation is found in MAG ZNRs, where the 100 and 101 peak intensities were the minimum. However, the worst ZNR orientation was found in Ga-doped ACG ZNRs sample. The crystalline quality and defect structure were confirmed by measuring the full width half maximum (FWHM) of the 002 peaks, which was further used to calculate the crystallite size of the samples via the Scherrer formula [25]:

(2) D=(0.89λ)/(B cosθ),

where *B* is the FWHM of the 002 peak in radians, *λ* is the X-ray wavelength, and *θ* is the 002 peak diffraction angle. Besides the crystallite size, the crystalline qualities of the samples were further checked by measuring the strain along the 002 phase. The strain (ε) was calculated by the relation:

(3) $$\varepsilon =\frac{C-C_{o}}{C_{o}}\times 100,$$

where *c_o_* is the lattice constant of stress-free bulk ZnO (5.205 Å). For stain calculation, the lattice constants (*c*) of the fabricated samples were measured by:

(4) $$c=\frac{\lambda }{sin\theta },$$

where *θ* corresponds the XRD 002 peak, and *λ* is the X-ray wavelength. The details of the structural characteristics of all the samples are provided in Table 1. It is evident that the D_MAG_ > D_OPT-ACG_ > D_Ga-doped ACG_ > D_ACG_, which confirms the deterioration in the crystalline structure in ACG and Ga-doped ACG samples. Similarly, MAG and OPT-ACG samples were less prone to strain than ACG and Ga-doped ACG samples. The negative sign in strain values implies that all of the samples encountered compressive strain rather than tensile. The yield stress (σ) in the samples could be calculated by the Hall–Petch equation:

(5) $$\sigma =\sigma_{0}+\frac{k}{\sqrt{D}},$$

where $\sigma_{0}$ and k are constants to the material that make the factor σy inversely proportional to the crystallite size D. Hence, the least stress was expected of the large crystallite size MAG sample, and the highest stress was encountered in least crystallite size ACG sample. Furthermore, the XRD results were in accordance to the PL results provided in the next section.

### 3.3. ZNR Photoluminescent Optical Characteristics

**Figure 4:**
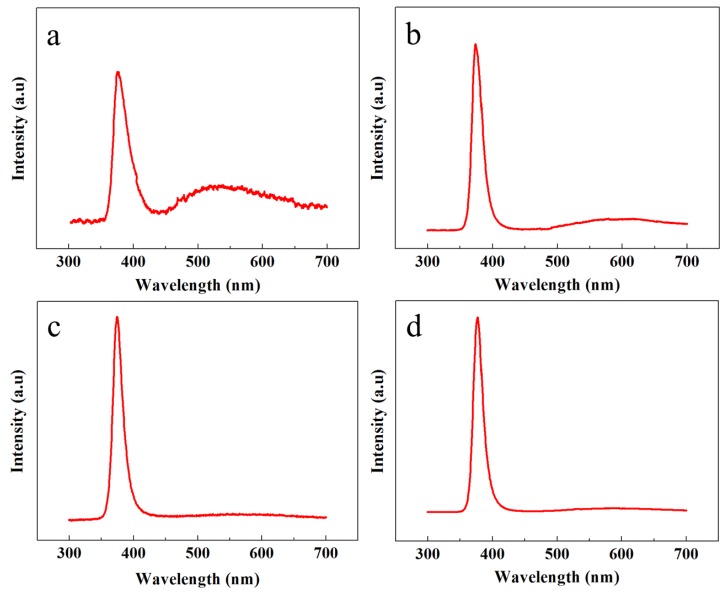
PL spectra of ZNRs grown with (**a**) ACG, (**b**) Ga-doped ACG, (**c**) OPT-ACG, and (**d**) MAG methods.

The crystalline structure, optical characteristics, and the presence of multiple defects were further classified with PL spectroscopy. Figure 4 shows the room temperature PL response of all the samples in the range of 300 to 700 nm. All the samples showed a high intensity peak in the UV region because of the exciton recombination process. However, the comparative intensity of the UV peaks and the UV-to-visible peak intensity ratio could be further exploited to test the crystalline structure, purity, and smooth optical character of the ZNRs. The highest UV peaks were shown by the OPT-ACG and MAG samples; however, the smallest UV peak was acquired from the ACG sample.

The defect engineering was verified by studying the PL responses in the visible region. It is evident that, depending upon the growth conditions, the PL peak intensities are different in the visible region. The heighest peak intensity was shown by the ACG sample, which confirms the presence of many defects in the crystal structure corresponding to the visible band. However, the peak intensity is reduced for the Ga-doped ACG sample, which confirms that Ga tends to fix many subsurface defects in the ZnO crystal [23]. On the contrary, the OPT-ACG and MAG samples show almost a flatband condition in the visible region, which confirms that the OPT process removes many surface and subsurface defects corresponding to the visible region, and the downward band bending in Figure 4c is the evidence to the reported fact. The highest UV peak intensity and the least visible peak intensity is shown by the MAG sample, which testifies the perfect ZNR crystal structure with best optical characteristics. The UV-to-visible band ratio (R) is an indicator of the ZNR crystal quality, where the greater value of R is analogous to a better crystalline structure. As per the values provided in Table 1, it is evident that R_MAG_ > R_OPT-ACG_ > R_Ga-doped ACG_ > R_ACG_, which is in accordance to the rest of the structural characteristics in Table 1. The PL optical and XRD structural characteristics established the base to explain the disparities in Au–ZnO contact behavior presented in the next section.

### 3.4. Growth Method-Dependent Defect Engineering

ZnO crystal quality and defect formation play a crucial role in Au–ZnO Schottky barrier engineering. ZnO has built-in properties of both covalent and ionic semiconductors, because the carrier transport and defect formation are quite complex in ZnO. The defect generation plays a crucial role in modulating the ZnO carrier concentration, because most of the ZnO defects are donor defects [26,27,28]. The topic is still very controversial, and this is a moot point in research and development, but oxygen vacancies are still considered to be one common defect that acts as an electron donor to ZnO. The theory for ambient hydrogen being used as a shallow donor to ZnO was first propounded by Mollwo, and substantiated by Van de Walle [29,30]. It is believed that hydrogen reacts with ZnO surface oxygen and forms OH^−^ ions at ambient growth conditions by donating an electron to the surface accumulation layer [31,32,33]. The process could be explained by the equation [34]:

H + O^2−^ → OH^−^ + e^−^ (6)

where O^2−^ is the surface-adsorbed oxygen via the chemisorption process, which is desorbed by H, and the depletion region is relaxed. In contact behavior studies, it is believed that the extrinsic factors, such as surface preparation and growth conditions, have a strong impact on the reduction of the Schottky barrier lowering. However, we believe that the intrinsic factors, such as defect formation, are directly dependent upon the extrinsic factors such as growth conditions. For instance, the thermal energy provided to the ZnO atoms to sit on proper crystallographic planes was not sufficient in low-temperature ZNR growth via the ACG method, and the process leads to the generation of many defects, and the vacant cites have been supplanted by donor impurities such as hydrogen [35]. Secondly, the existence of the sample in the growth solution for a long time (12 hr in the ACG method) paves the way for impurity adsorption on the ZNR surface. On the contrary, the OPT process removes the adsorbed impurities on the ZNR surface, and high-power MAG provides the energy required by the atoms to nucleate from the proper crystal sites, and both processes lead to the least generation of defects. Hence, the extrinsic factors directly affect the generation of defects that modulate the fermi level position and Schottky barrier pinning. Because of defect density-induced Schottky barrier pinning, the effective barrier height as well as width at the Au–ZnO interface would be changed, which supports Ohmic/Schottky behaviors to ZnO. Furthermore, some defects are introduced into the ZnO bandgap to support electron hopping through the barrier.

### 3.5. Au–ZnO Contact Behavior

Depending upon the base provided in Section 3.4, the Au–ZnO contact behavior could be divided into multiple sections and is provided in the next section.

#### 3.5.1. Au–ZnO Ohmic Contact to ZNRs

**Figure 5:**
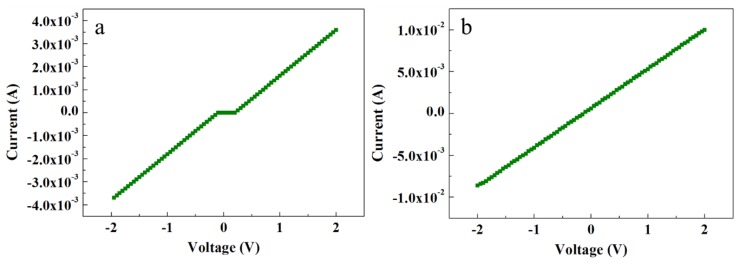
*I*-*V* characteristics of (**a**) ACG ZNRs and (**b**) Ga-doped ACG ZNRs.

Figure 5 shows the electrical characteristics of ACG and Ga-doped ACG samples. It is evident that the ACG sample shows a quasi-Ohmic response, while Ga-doped ACG sample shows a more pronounced Ohmic response to the Au contact. The reason for this unusual response to Au is the presence of many surface and subsurface defects in ACG ZNRs, as explained earlier in Section 3.4. Also, further impetus was provided by the formation of an accumulation layer via hydrogen adsorption, which denounces the formation of the Schottky barrier at the ZNR surface via the chemisorption process. This leads to the Schottky barrier pinning, where the barrier height and width are decreased at the interface. The relation between the tunneling energy “E” and the carrier concentration “N” is governed by the equation [36]:

(7) $$E=\frac{qh}{4\pi }\frac{\sqrt{N}}{\sqrt{m\varepsilon_{o}\varepsilon_{s}}}$$

where *h* is the Plank constant, *m* is the effective mass, and *ε*_s_ is the ZnO dielectric constant. It is evident in Equation (7) that the tunneling energy *E* is directly proportional to the factor $\sqrt{N}$. The average carrier concentrations in the ACG and Ga-doped samples were measured via a Hall-effect measurement setup, which was of the order of 10^18^ and 10^21^ cm^−3^, respectively. Hence, the carrier transport in ACG and Ga-doped ACG samples was governed by thermionic emission by reducing the Schottky barrier height, field emission through the thin barrier, and thermionic field emission near the top of the barrier. At high voltages, the deep-level defects could be further supported by electron hopping within the forbidden band. To increase the carrier concentration, the ACG sample was further doped with Ga. The doped sample showed a more pronounced Ohmic contact to Au with a highest intensity current, as shown in Figure 5b. The Fermi level position in bulk ZnO was predicted to be 0.3 eV under the conduction band [37]. However, we believe that the Fermi level position that was very close to the conduction band and diffusion into the conduction band in ACG and Ga-doped ACG samples, respectively, was the reason for these Au–ZnO quasi-Ohmic (ACG) and Ohmic contacts (Ga-doped ACG). The Schottky barrier height is governed by:

(8) $$q\varphi_{SB}={\mathrm{qV}}_{B}+(E_{c}-E_{f})$$

where the quantity (*E_c_* − *E_f_*) < 0 for ACG and Ga-doped ACG samples at the interface and the effective value of *φ_SB_* was decreased.

#### 3.5.2. Au–ZnO Schottky Contact to ZNRs

**Figure 6:**
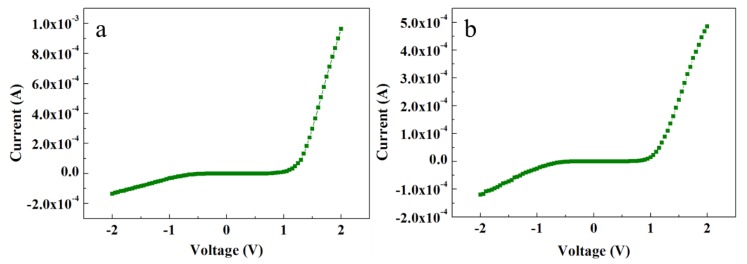
*I*-*V* characteristics of (**a**) OPT-ACG ZNRs and (**b**) MAG ZNRs.

Contrary to the ACG and Ga-doped ACG samples, the OPT-ACG and MAG samples showed Au–ZnO Schottky contacts. Figure 6a,b show the *I*-*V* characteristics of the OPT-ACG and MAG samples, respectively. Both samples show Schottky-type *I*-*V* characteristics because of the decrease in the carrier concentration-induced increase in Schottky barrier height and the barrier broadening at the interface. The OPT process was used as a ZNR surface cleanser, where it removed the hydrogen from ZNR surface, which is a source of donor electrons. Furthermore, the accumulation layer formed on the ZNR surface near the Au–ZnO interface was also removed in the OPT-ACG and MAG samples, which provided further impetus to the chemisorption process and strengthened the interface Schottky barriers in both samples. Zn vacancies act as deep acceptors, and oxygen vacancies are the primary sources of deep donors in ZnO. The OPT process did not affect the Zn-acceptor vacancies, but filled the potential sites for donor oxygen vacancies, reducing the overall n-type carrier concentration in the ZNR channel. It also removed the hydrocarbon contamination from the surface, which aided in the defect formation process. As explained in Section 3.4, the immaculate nature of MAG method grew clean and low-defect ZNRs with the least carrier concentration. A reduction in visible band intensity in PL spectra (see Figure 4c,d) is the evidence to the above-mentioned phenomenon. Hence, the MAG and OPT processes did not only eliminate the OH^–^ ion inserted to the surface accumulation layer, but also reduced the deep level defects to eliminate the effects of processes such as tunneling, depletion-width thinning, and hopping. As per Equation (7), the tunneling energy E is the minimum in OPT-ACG and MAG samples, hence, the carrier transport is only governed by thermionic emission at high voltage levels. Also, the factor *E_c_* − *E_f_* was greater than zero because of the Fermi level shifting below the conduction band, which further strengthened the Schottky barriers (*φ_SB_*) as per Equation (8).

#### 3.5.3. Au Deposition Process Details

At times, the metallization process also incorporates many defects into the ZnO crystal, which further supports field emission and thermionic field emission processes. The metallization chemistry and phenomenon is complicated, and is still a matter of debate in research and development on ZnO metallization schemes. The metallization process has the probability of dislodging many oxygen atoms from the crystal structure which move towards the metal surface via diffusion. The out-diffusion of oxygen atoms leaves behind oxygen vacancies that are donor defects to ZnO, and further supports tunneling near the metal-semiconductor surface. However, we believe that it was not the case in our devices’ metallization process, because at first, the metal was evaporated via a state of the art e-beam metal evaporation process under high-vacuum conditions. Secondly, Au is one of the inert metals that does not incorporate defects at the Au–ZnO interface via oxygen vacancies’ out-diffusion, and supports barrier pinning. Also, it does not react with ambient oxygen to form an insulating oxide layer at the Au–ZnO interface to support barrier width broadening. Hence, the propounded results are purely on the basis of defect engineering rather than any complementary processes.

## 4. Conclusions

In this study, the disparities in Au–ZnO contact behavior were probed via experimentation and results. The growth method-induced defect engineering was cited as the main reason for the said disparities. Two antithetical growth methods, namely ACG and MAG, were used for ZNR growth. The ACG samples were further doped with Ga and subjected to OPT to probe into the subsurface and surface-level defect engineering-centric change in carrier concentration, and its effects on Au–ZnO Schottky barrier heights. Also, the ZNR morphology and dimensions were standardized to study the change in Au–ZnO contact behavior because of defects/carriers, rather than the change in surface-to-volume ratio. The XRD and PL results qualified that the MAG sample had the best crystalline quality and optical character. It was further argued that the OPT process removed many surface and subsurface level defects in the ZnO crystal, which improved the ZNR crystalline structure and optical characteristics. Despite having a theoretical Schottky barrier at the Au–ZnO interface, the ACG and Ga-doped ACG samples showed a quasi-Ohmic and Ohmic behaviors, respectively. This was because of the defects and doping-induced increase in carrier concentration in the ZNR channel and the Schottky barrier pinning, which facilitated tunneling through the thin barrier. Contrarily, the defect scanty OPT-ACG and MAG samples further strengthened the Schottky barriers at the Au–ZnO interface and showed Schottky contacts to ZnO. Hence, the reason for the change in Au–ZnO contact behavior lies in the method-dependent defect engineering, which needs to be modulated for the specified use of contact in application-oriented research and development on Au–ZnO.

## Figures and Tables

**Table 1:** Structural and optical parameters of all the samples.

Sample	θ (Deg.)	FWHF (Rad.)	D (nm)	c (Å)	ε (%)	R
ACG	17.28	0.0048	30.44	5.184	−0.403	03.33
Ga-doped ACG	17.26	0.0047	31.13	5.185	−0.384	25.00
OPT-ACG	17.24	0.0033	44.19	5.202	−0.057	47.12
MAG	17.23	0.0024	62.27	5.203	−0.038	58.15
